# Mud Loss Restricts Yki-Dependent Hyperplasia in *Drosophila* Epithelia

**DOI:** 10.3390/jdb8040034

**Published:** 2020-12-13

**Authors:** Amalia S. Parra, Christopher A. Johnston

**Affiliations:** Department of Biology, University of New Mexico, Albuquerque, NM 87131, USA; sancham9@unm.edu

**Keywords:** Yorkie, Mud, collagen, wing disc, Drosophila, hyperplasia, transcriptome

## Abstract

Tissue development demands precise control of cell proliferation and organization, which is achieved through multiple conserved signaling pathways and protein complexes in multicellular animals. Epithelia are a ubiquitous tissue type that provide diverse functions including physical protection, barrier formation, chemical exchange, and secretory activity. However, epithelial cells are also a common driver of tumorigenesis; thus, understanding the molecular mechanisms that control their growth dynamics is important in understanding not only developmental mechanisms but also disease. One prominent pathway that regulates epithelial growth is the conserved Hippo/Warts/Yorkie network. Hippo/Warts inactivation, or activating mutations in Yorkie that prevent its phosphorylation (e.g., Yki^S168A^), drive hyperplastic tissue growth. We recently reported that loss of Mushroom body defect (Mud), a microtubule-associated protein that contributes to mitotic spindle function, restricts Yki^S168A^-mediated growth in *Drosophila* imaginal wing disc epithelia. Here we show that Mud loss alters cell cycle progression and triggers apoptosis with accompanying Jun kinase (JNK) activation in Yki^S168A^-expressing discs. To identify additional molecular insights, we performed RNAseq and differential gene expression profiling. This analysis revealed that Mud knockdown in Yki^S168A^-expressing discs resulted in a significant downregulation in expression of core basement membrane (BM) and extracellular matrix (ECM) genes, including the type IV collagen gene *viking*. Furthermore, we found that Yki^S168A^-expressing discs accumulated increased collagen protein, which was reduced following Mud knockdown. Our results suggest that ECM/BM remodeling can limit untoward growth initiated by an important driver of tumor growth and highlight a potential regulatory link with cytoskeleton-associated genes.

## 1. Introduction

Tissue development requires proper assembly of three-dimensional shape and expansion to the correct final size. Loss of tissue architecture and improper growth regulation underlies numerous developmental disorders and is a hallmark feature of cancer. Thus, deciphering the genetic basis for these complex events and the underlying regulatory mechanisms involved are critical endeavors. Epithelia are a ubiquitous tissue type that line inner and outer surfaces of major organ systems throughout the body. Epithelial cells adopt distinct shapes and organizations that contribute to their diverse functions. Disruption of their discrete architectures can lead to tissue dysfunction and abnormal growth [1]. Notably, epithelia are a common driver of tumorigenesis in many organs (e.g., carcinomas) and these tumors often arise following mutations in genes controlling growth and proliferation, as well as in those involved in polarity and junctional organization [2]. It is therefore important to continue advancing our understanding of the complex dynamics of epithelial growth, including in model organism systems.

*Drosophila* imaginal discs are an excellent, well-established genetic system for studying epithelia development and disease [3]. These sac-like structures present in larvae consist of columnar and squamous epithelial cells and transform into external structures (e.g., wings) during pupal development [4]. Imaginal discs display many features of typical epithelial tissue during development including high proliferation rate, formation of specialized cell-cell junctions, establishment of cell and tissue polarity, and planar orientation of cell divisions, yet their relatively simple structure is ideally suited for genetic and imaging studies [5]. Importantly, many of the genes that regulate imaginal disc development and growth are highly conserved in humans, including in the wing disc model used in this study.

One signaling network with considerable importance to epithelial growth dynamics is the evolutionarily conserved Hippo/Yorkie pathway. Hippo kinase (human Mst1/2), together with the scaffold Salvador, phosphorylates and activates Warts kinase (human LATS1), which in turn phosphorylates the transcriptional regulator Yorkie (Yki; human YAP/TAZ). Then, 14-3-3 proteins bind and sequester phosphorylated Yki in the cytoplasm, thus preventing nuclear activation of growth-promoting genes [6]. In this paradigm, the Hippo/Warts complex serves as a critical tumor suppressor and studies have shown that loss-of-function in these genes yields excessive tissue growth [7]. Moreover, hyperactivation of Yki is commonly associated with human tumors and represents an attractive target for cancer treatment [8]. Similar effects have been extensively modeled in *Drosophila*, with Hippo/Warts dysfunction or Yki hyperactivation leading to amplified tissue growth [9]. In wing discs, such mutations alone lead to hyperplastic growth, characterized by overgrown tissue that retains normal epithelial polarity and cell–cell junctions [10]. Recent studies have identified additional mutations that genetically interact with Yki, several of which lead to oncogenic transformation and neoplastic growth following a breakdown of such tissue organization [11,12,13,14,15,16]. Indeed, an RNAi-based screen recently identified numerous genes whose concomitant knockdown transformed Yki-mediated growth to neoplastic tumors [12]. Owing to its essential role in tissue growth and development, it is important to identify and understand how additional signaling pathways and cellular components intersect with Yki-dependent growth, particularly in identifying those whose inactivation can suppress rather than transform such hyperplasia.

We recently found that knockdown of Mushroom body defect (Mud), a conserved microtubule (MT)-associated protein involved in mitotic spindle assembly and function, suppresses hyperplastic wing disc growth induced by expression of constitutively active Yki^S168A^ [17], which is rendered insensitive to Warts phosphorylation in the key 14-3-3 binding motif [18]. This previous work also identified a role for Warts kinase in Mud-dependent spindle positioning, suggesting Hippo signaling regulates both the rate and orientation of cell divisions within wing disc epithelia [19]. Here, we sought to identify molecular mechanisms through which Mud loss attenuates Yki^S168A^-dependent growth. We found that Mud knockdown altered cell cycle progression and triggered apoptosis, with Yki^S168A^-expressing discs being hypersensitive to this effect. This response was accompanied by activation of Jun kinase (JNK), suggesting apoptosis is JNK-dependent. We also performed whole transcriptome sequencing and differential gene expression analysis, which found that Mud knockdown leads to alteration in expression of hundreds of genes. Interestingly, a subset of genes specifically downregulated following Mud loss in Yki^S168A^ discs belong to the collagen and related families of basement membrane (BM) and extracellular matrix (ECM) genes. Immunostaining against collagen proteins revealed that Yki^S168A^-expressing discs accumulate significantly more of this core BM component, and that Mud knockdown reduces this effect. Our findings highlight a novel genetic interaction between a conserved cytoskeletal regulator and Yki-driven growth in a model epithelial tissue and suggest a role for ECM/BM remodeling in controlling hyperplastic proliferation.

## 2. Materials and Methods

### 2.1. Drosophila Stocks and Maintenance

Unless noted, all fly stocks used in this study were obtained from the Bloomington *Drosophila* Stock Center and were as follows: *nubbinGAL4* (#25754), *UAS:mud^RNAi^* (#35044), *UAS:Yki^S168A^* (#28818), and *nubbin^GAL4^;UAS:mud^RNAi^* [17]. Stocks were maintained at 25 °C, and genetic crosses were raised at 29 °C on food containing 0.05% bromophenol blue for all experiments (to facilitate identification and staging of third instar larvae for selection), unless otherwise noted.

### 2.2. Antibody Staining

Imaginal wing discs from staged third instar larvae were dissected in ice-cold PBS followed by fixation for 23 min in 4% paraformaldehyde. Tissues were washed three times for 10 min in PBS-T (1× PBS, 0.3% Triton) and blocked for 1 h at room temperature using PBS-BT (1× PBS, 0.3% Triton, 2.5% goat serum, 2.5% donkey serum), then incubated overnight in primary antibody solution diluted with PBS-BT at 4 °C. Following this incubation, tissues were washed three times for 20 min in PBS-BT followed by incubation in secondary antibody diluted in PBS-BT overnight at 4 °C. Imaginal wing discs were mounted in 80% glycerol and stored at 4 °C until imaging.

The following antibodies were used: rabbit cleaved caspase-3 (CC3) (1:500, Cell Signal, #9661S), phalloidin-568 (1:50, ThermoFisher, #A12380), rabbit phosphorylated JNK (pJNK) (1:1000, Promega, #V7932), rabbit phosphohistone-H3 (PH3) (1:1000, ThermoFisher, #PA5-17869), and mouse type IV collagen (1:50, DSHB).

### 2.3. Edu Staining

Imaginal wing discs from staged third instar larva were dissected in PBS followed by incubation for 30 min in 100 ug/mL 5-Ethynyl-2′-deoxyuridine (EdU) at room temperature. Following incubation, tissues were processed for antibody staining as described above, and EdU detection was performed prior to incubation in secondary antibody using a Click-iT EdU Cell Proliferation Kit 488 (Thermofisher, #C10337, Waltham, MA) and accompanying protocol.

### 2.4. Image Acquisition and Processing

Images were acquired using a Zeiss LSM-780 confocal microscope (Carl Zeiss Microscopy, White Plains, NY, USA). Analysis was performed using Fiji software (v1.53 open source) and figures were assembled in Adobe Illustrator. Area quantification and percent area positive for EdU, PH3, and pJNK of wing disc maximum intensity projections were done using thresholding and the ”thresholdcolour” plugin in ImageJ. The area of the wing pouch was first taken using the polygon selection tool and recorded in pixels. The extraneous portions of the disc (the hinge and the notum that lie outside of the Nubbin expression pattern) were then removed using the ”Clear Outside” command. Then, using the ”Threshold Colour” command, only pixels displaying green were selected. Using the ”Threshold” command, pixels positive for signal were selected and background was excluded. The number of positive pixels was then calculated using the ”Analyze Particles” command, with the minimum detectable pixel size set to 2 square pixels, and the results displayed. The number of pixels obtained was then normalized to a percent area measurement by dividing it by the size of the wing pouch obtained earlier.

Intensity quantification of type IV collagen staining was performed by converting images to gray scale and selecting the region of interest (e.g., wingpouch) using the polygon tool, followed by thresholding. The intensity of pixels within the thresholded area was subsequently calculated using the measurement function and plotted as an average pixel intensity across the selected tissue.

### 2.5. RNA Sequencing

RNA sequencing was performed on three biological replicates using the Illumina Next Generation Sequencing platform (Illumina, San Diego, CA, USA). Libraries were prepared using 500 ng total RNA and a KAPA mRNA Hyper Prep kit (Roche, Indianapolis, IN). Raw reads were trimmed and filtered using Trimmomatic v0.36 with a slide window of 4 nt, average score above 20 and minimum length of 36 nt [20]. High-quality reads were mapped to the *D. melanogaster* genome (NCBI version GCA_000001215.4 Release 6 plus ISO1 MT) using STAR v2.5.3a (Cold Spring Harbor Laboratory) [21]. Transcripts expression levels were estimated using featureCounts v1.6.2 (Subread, General Public License) and differential gene expression analysis was performed using EBSeq v1.18.0 [22]. Genes with an adjusted *p* ≤ 0.05 and log_2_-fold change >1 (upregulated) or < 1 (downregulated) were considered for further analysis. Gene ontology (GO) and pathways analyses were conducted using DAVID software v6.8 [23,24], and motif analyses were performed using FIMO in MEME suite [25].

## 3. Results and Discussion

### 3.1. Mud Knockdown Attenuates Yki^S168A^-Driven Wing Disc Growth

**Figure 1:**
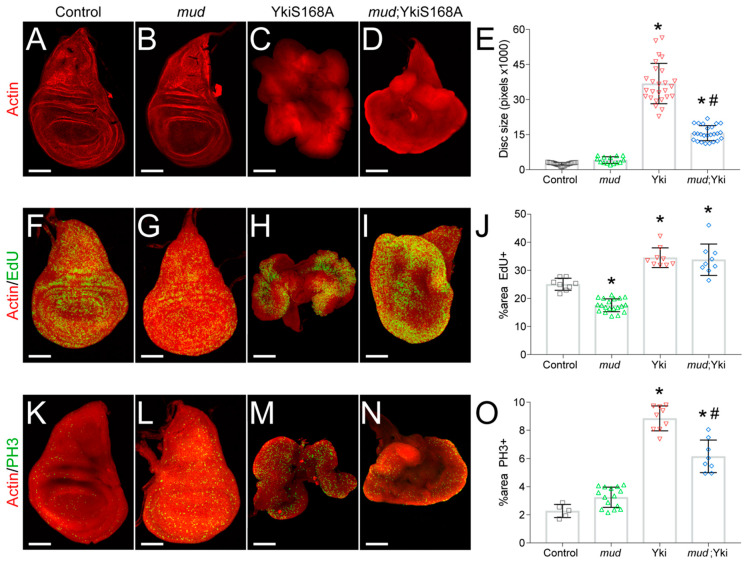
*mud^RNAi^* alters cell cycle progression and restricts growth in Yki^S168A^-expressing imaginal wing discs. (**A**–**D**) Phalloidin staining of F-actin shows size of imaginal wing discs dissected from late third instar larvae of the indicated genotype. Images shown are representative of at least 15 discs. (**E**) Bar graph depicts the average ± standard deviation of wing disc pouch area measured in total pixels. *, *p* < 0.05 compared to Control; #, *p* < 0.05 compared to Yki^S168A^ alone, ANOVA with Tukey’s post-hoc test. (**F**–**J**) EdU staining marks actively proliferating cells, with phalloidin co-stain to show disc morphology. Images shown are representative of at least 10 discs. (**J**) Bar graph depicts the average ± standard deviation of EdU^+^ cells measured in percent of total wing disc pouch. *, *p* < 0.05 compared to Control; #, *p* < 0.05 compared to Yki^S168A^ alone, ANOVA with Tukey’s post-hoc test. (**K**–**N**) PH3 staining marks mitotic cells, with phalloidin co-stain to show disc morphology. Images shown are representative of 5–20 discs. (**O**) Bar graph depicts the average ± standard deviation of PH3^+^ cells measured in percent of total wing disc pouch. *, *p* < 0.05 compared to Control; #, *p* < 0.05 compared to Yki^S168A^ alone, ANOVA with Tukey’s post-hoc test. Note that images are not shown at identical scales. Specifically, scale bars shown represent 50, 50, 250, and 150 microns for Control, *mud*, Yki^S168A^, and *mud*;Yki^S168A^ images, respectively.

Nuclear translocation of Yki activates expression of many genes involved in cell proliferation and inhibition of apoptosis, ultimately serving as a key size control pathway during tissue development [26,27]. The phosphorylation insensitive Yki^S168A^ is a constitutively active mutant and its overexpression is sufficient to induce tissue overgrowth in both humans and *Drosophila*, including imaginal discs [28]. Whereas Yki^S168A^ expression alone induces wing disc hyperplasia, synthetic interactions with several other gene mutations can result in transformation to neoplastic growth [11,14,15]. In contrast, we recently found that knockdown of Mud through expression of small hairpin interfering RNA (*mud^RNAi^*) using the *nubbin^GAL4^* driver reduces hyperplastic wing disc growth in response to the hyperactive Yki^S168A^ mutant [17], a result that we have replicated here (Figure 1A–E). Specifically, Yki^S168A^ expression induced a ~15-fold increase in wing disc size in late L3 stage larvae, which was significantly reduced by *mud^RNAi^* expression. In contrast, Mud knockdown alone did not significantly alter disc size (Figure 1B,E). Note that in all figures discs are not necessarily shown at the same scale; specific scale bar measurements are listed in the figure captions. Representative images of discs scaled identically, which more clearly show the size differential, can be seen in Figure S1 of the supplementary materials.

To understand the molecular basis for this effect, we first examined how Mud knockdown impacts proliferation and cell cycle progression. To determine the percentage of cells actively entering the cell cycle, we stained wing discs with the thymidine analog 5-Ethynyl-2′-deoxyuridine, which is incorporated into DNA of proliferating cells. As expected, hyperplastic Yki^S168A^-epxressing discs showed an increased EdU^+^ index (Figure 1H,J). In contrast, *mud^RNAi^* expression by itself caused a decrease in EdU^+^ cells (Figure 1G,J). Mud knockdown failed, however, to reduce the EdU^+^ index in Yki^S168A^ discs (Figure 1I,J), indicating that the proliferative effects of Yki activation persist despite *mud^RNAi^* expression. We next assessed the mitotic index in wing discs using the phosphohistone-3 label of condensed mitotic chromosomes, thus marking entry into the M-phase from G2. Similar to EdU, we observed an increase in PH3^+^ cells following expression of Yki^S168A^ (Figure 1M,O), a result consistent with Yki activation in other tissues [29,30]. Mud knockdown alone resulted in a modest increase in PH3 staining (not significant); however, it caused a statistically significant reduction in the PH3^+^ mitotic index induced by Yki^S168A^ expression (Figure 1K–O). Thus, while Mud knockdown does not affect the increased percent of actively proliferating cells caused by Yki^S168A^ expression, it does appear to alter cell cycle progression. More specifically, the decrease in PH3 staining (compared with no change in EdU) is indicative of a failure to enter mitosis and possible G2 phase stalling or arrest. A mechanism for this effect is not currently clear, although several cellular stressors and responsive signaling pathways have been implicated in G2 arrest [31,32].

### 3.2. Mud Knockdown Induces JNK Activation and Apoptosis in Yki^S168A^-Expressing Wing Discs

**Figure 2:**
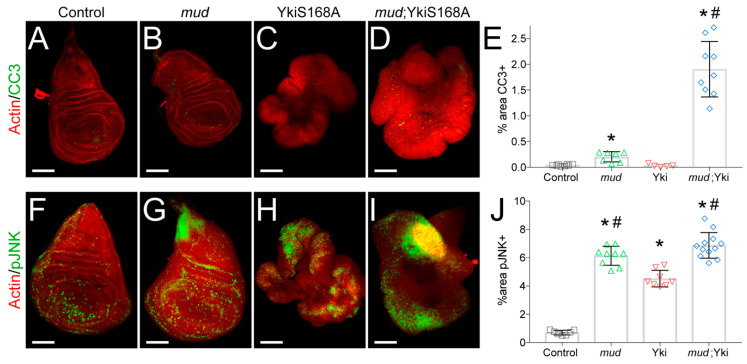
*mud^RNAi^* triggers JNK activation and apoptosis in Yki^S168A^-expressing imaginal wing discs. (**A**–**D**) Cleaved caspase-3 (CC3) staining marks apoptotic cells, with phalloidin co-stain to show disc morphology. Images shown are representative of 5–10 discs. (**E**) Bar graph depicts the average ± standard deviation of CC3^+^ cells measured in percent of total wing disc pouch. *, *p* < 0.05 compared to Control; #, *p* < 0.05 compared to Yki^S168A^ alone, ANOVA with Tukey’s post-hoc test. (**F**–**I**) Phosphorylated JNK (pJNK) staining marks cells with activated JNK, with phalloidin co-stain to show disc morphology. Images shown are representative of 5–15 discs. (**J**) Bar graph depicts the average ± standard deviation of pJNK^+^ cells measured in percent of total wing disc pouch. *, *p* < 0.05 compared to Control; #, *p* < 0.05 compared to Yki^S168A^ alone, ANOVA with Tukey’s post-hoc test. Note that images are not shown at identical scales. Specifically, scale bars shown represent 50, 50, 250, and 150 microns for Control, *mud*, Yki^S168A^, and *mud*;Yki^S168A^ images, respectively.

In addition to its effects on cell proliferation, Yki also suppresses apoptosis through transactivation of the *Drosophila inhibitor of apoptosis-1* gene (*diap1*) [7]. Together with the apparent effects of Mud knockdown on cell cycle progression in Yki^S168A^-expressing discs (Figure 1), this led us to next examine how *mud^RNAi^* expression affects cell survival. As shown in Figure 2, Mud knockdown alone in a wild-type background induced a modest increase in apoptosis marked by cleaved caspase-3. This result is in agreement with recent studies demonstrating that loss of other mitotic spindle regulatory genes induces cell death in larval wing discs [33,34,35,36,37]. Strikingly, *mud^RNAi^*-induced apoptosis was significantly enhanced in Yki^S168A^-expressing discs (Figure 2D,E). These results demonstrate that Yki^S168A^ discs are hypersensitive to Mud loss and suggest that cell death contributes to their limited growth following loss of this key mitotic regulator.

Recent studies have identified a complex role for Jun kinase activation in wing disc growth as well as cell death [33,34,35,36,37]. Furthermore, JNK-induced apoptosis is antagonized by Yki signaling during both normal development and tumorigenesis [11,38]. To assess the role of JNK signaling, we stained wing discs for active, phosphorylated JNK. We found that both *mud^RNAi^* and Yki^S168A^ expression increased the percentage of cells positive for pJNK staining, with Mud knockdown inducing a greater percentage of the disc (Figure 2F–J). pJNK levels in *mud^RNAi^*; Yki^S168A^ double mutants were indistinguishable from *mud^RNAi^* alone discs (Figure 2F,J), indicating that Mud knockdown exacerbates JNK activation in discs with activated Yki. We also analyzed the average intensity of the pJNK signal and found these same trends across the four genotypes, suggesting this response is both stronger and more widespread throughout the disc (data not shown). The seemingly paradoxical effects of JNK signaling on wing disc growth are complex, and whether this pathway ultimately triggers cell proliferation or apoptosis appears to depend on other cellular contexts [39]. Our results suggest that the elevated apoptosis following *mud^RNAi^* expression is likely JNK-mediated in both control and Yki^S168A^ backgrounds, and may involve at least a partial switch from a proliferation signal in the latter [40]. JNK signaling regulates the G2/M checkpoint in human cell lines [41,42] and, interestingly, has recently been shown to induce cell cycle stalling in the G2 phase in wing disc cells [43], a result consistent with results shown above. Thus, *mud^RNAi^* expression leads to JNK activation that likely underlies its ability to trigger apoptosis.

### 3.3. Mud Knockdown Alters the Transcriptional Landscape of Wing Disc Epithelial Cells

**Figure 3:**
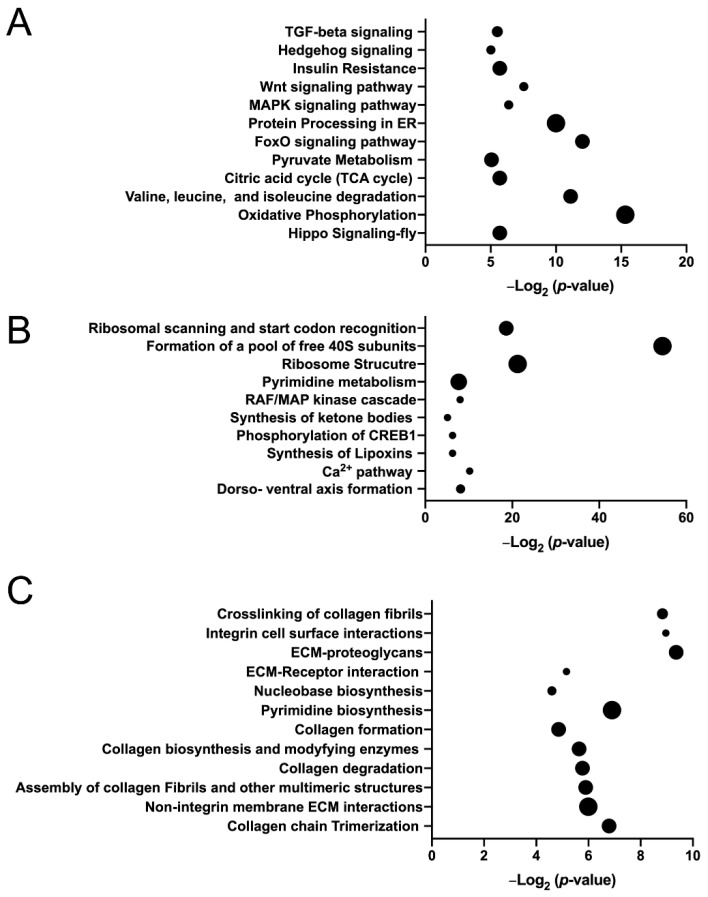
Pathway analysis of differentially-expressed genes following *mud^RNAi^* and Yki^S168A^ expression in imaginal wing discs. (**A**) Dot plot shows enriched pathways (*p* < 0.05) identified using DAVID for Yki^S168A^ discs compared to Control. The size of the dot corresponds to the number of differentially-expressed genes enriched in each listed pathway. (**B**) Dot plot shows enriched pathways (*p* < 0.05) identified using DAVID for *mud^RNAi^* discs compared to Control. The size of the dot corresponds to the number of differentially-expressed genes enriched in each listed pathway. (**C**) Dot plot shows enriched pathways (*p* < 0.05) identified using DAVID for *mud^RNAi^*; Yki^S168A^ discs compared to Yki^S168A^ alone. The size of the dot corresponds to the number of differentially-expressed genes enriched in each listed pathway. In each panel, dot sizes represent gene clusters that increase by five genes per incremental dot size.

Having demonstrated that JNK activation and apoptosis, both commonly seen following defects in other spindle-regulating genes [11,33,35], are concomitant with restricted Yki^S168A^ growth in response to *mud^RNAi^* expression, we next sought to identify additional, potentially novel aspects of the interplay between Mud and Yki. To do this, we performed RNAseq-based whole transcriptome sequencing and differential gene expression analysis on wing disc pouches dissected from late third instar larvae (Table S1). Yki^S168A^ expression resulted in 2155 upregulated and 2143 downregulated genes relative to control wing discs (Table S2). Pathway analysis found several notable signal transduction pathways involved in tissue growth and development, including TGF-β, Hedgehog, MAP kinase, Wnt, and Hippo pathways (Figure 3A). Additional pathways identified correlate with cell metabolism, including oxidative phosphorylation. Enriched gene ontology terms are listed in Table 1 and include several processes related to metabolism and ribosome activity. Among the genes upregulated in Yki^S168A^ discs were the well-established Hippo pathway targets *myc*, *diap1,* and *ex* (the *yki* transcript level was also highly upregulated as expected), consistent with UAS-mediated Yki overexpression [29,44,45]. Numerous additional genes implicated in Yki activity were also found, including *kibra, cher, ftz-f1, jbug, sog, ilp8, merlin, sav, jub, wts,* and *tai* [46]. Among the most highly upregulated genes was a group of BTB-zinc finger domain genes comprised of *chinmo, fruitless, abrupt,* and *ken,* as well as *lola* to a less significant degree. Interestingly, these genes are JNK targets and necessary for both Ras- and Notch-induced epithelial hyperplasia and tumorigenesis [47,48,49]. Lastly, numerous ECM and ECM-related genes, members of the so-called ”matrisome” [50,51], were identified in the upregulated pool of genes, whereas other member genes were among those downregulated (Table 2; also see below for further discussion). Further examination of downregulated genes identified key members of the *Drosophila* cell death pathway (e.g., *hid, rpr, dronc, drice,* and *dcp-1*), consistent with reduced apoptotic efficiency. Also found were numerous genes previously identified as targets of Yki-mediated transcriptional repression, including *elav*, *eya*, *dac*, and *wg* among others [52,53]. Overall, we found consistent agreement between Yki^S168A^-indcued transcriptional changes and those identified previously in wing discs using loss-of-function mutation in *wts*, the key Yki inhibitor [54,55], which share similar profiles with other epithelial tumor drivers, such as Ras and Notch [46].

In contrast to Yki expression, the effects of Mud knockdown on gene expression have not been studied previously, although the transcriptional changes following knockdown of SAS-4, another spindle pole associated gene, have recently been reported [36]. Similarly, we found that *mud^RNAi^* expression caused a significant impact on the wing disc gene expression profile. Specifically, Mud knockdown resulted in 291 upregulated and 327 downregulated genes relative to control discs (Table S2). Pathway analysis of these genes identified ribosome structure, cell metabolism, and regulation of the CREB transcription factor among those most affected (Figure 3B). Furthermore, transcription factor motif analysis using FIMO identified 77 putative CREB target genes (12.5% of total; see Table S3) in the pool of differentially expressed genes in the *mud^RNAi^*-expressing discs, which contained a total of 238 motifs [25,56]. Additional analysis identified 219 putative target genes (35.4% of total; see Table S3) for Jun, the transcription factor target of activated JNK, consistent with the pJNK staining (Figure 2G,J). Thus, Mud knockdown appears to utilize Jun and CREB as potential effectors for its transcriptional response. Enriched gene ontology terms are listed in Table 3 and included RNA-binding and translation processes among others. These GO terms differ rather dramatically from those reported in SAS-4 mutants [36], suggesting that wing discs mount unique responses to disruption of distinct spindle-regulating genes. Importantly, the *mud* transcript level itself was significantly downregulated, an expected outcome following *UAS:mud^RNAi^* expression and consistent with its efficacy. The precise role for these changes i disc response to Mud knockdown will require future study. However, we next focused more closely on the molecular interaction between *mud^RNAi^* and Yki^S168A^ expression.

To understand how Mud knockdown impacts gene expression specifically in Yki^S168A^-expressing discs, we evaluated differentially-expressed genes in *mud^RNAi^*;Yki^S168A^ discs compared to Yki^S168A^ alone. Here, Mud knockdown resulted in fewer changes relative to its comparison against control wing discs, with 31 upregulated and 37 downregulated genes compared to Yki^S168A^ expression alone (Table S2). Notably, this analysis revealed that *mud^RNAi^* expression did not result in altered expression of known Yki target genes, suggesting Mud knockdown does not directly affect Yki nuclear activity per se. Among the GO terms identified for genes upregulated in the *mud^RNAi^*;Yki^S168A^ discs were those involved in the regulation of cell death (Table 4), consistent with the cell cycle and apoptosis effects measured above (Figure 1 and Figure 2). Examination of downregulated genes in *mud^RNAi^*;Yki^S168A^ discs identified an abundance of genes involved in BM and ECM construction, which was reflected in the enriched GO terms (Table 4) as well as in the pathways analysis (Figure 3C). Several genes involved in formation of the wing cuticle, and its connection with the ECM, were also downregulated in *mud^RNAi^;yki^S168A^* discs [57]. Thus, whereas Yki^S168A^ expression upregulated numerous ECM component and regulatory genes, Mud knockdown downregulated distinct core ECM-related genes in Yki-expressing discs. These findings suggest that ECM/BM remodeling may play an important role in the growth patterns of these discs, particularly the growth restriction imposed by *mud^RNAi^* in a hyperplastic, Yki^S168A^-expressing background.

The ECM is a complex network of collagen and other glycoproteins that provides structural support as well as intercellular signaling in multicellular animals. The BM is a thin, specialized layer of the ECM that connects the epithelium with underlying connective tissue [58]. Among the downregulated genes identified in *mud^RNAi^;yki^S168A^* discs, the *Drosophila* type IV collagen gene *viking* (*vkg*) was particularly intriguing. Type IV collagen is the principal component of the BM and defects in its function have been associated with developmental and autoimmune disorders as well as cancer progression in humans [59,60]. In *Drosophila*, Vkg is secreted by hemocytes, essential cells of the innate immune system [61], and contributes to morphogenic development of diverse tissue types, including wing discs [62,63]. Moreover, recent studies have found that disruption of the BM through Vkg depletion leads to reduced wing disc size [64]. Consistent with this, all ECM genes including *vkg* identified in our analysis were downregulated specifically in *mud^RNAi^;yki^S168A^* discs and not following *mud^RNAi^* expression alone in otherwise normal discs. *Vkg* transcript levels did not statistically differ among other conditions relative to control, suggesting the reduction seen in *mud^RNAi^;yki^S168A^* discs represents a unique interaction between the *mud* and *yki* genes. Overall, we conclude that Mud knockdown alters epithelial cell transcriptome, including changes that implicate possible ECM and BM remodeling.

### 3.4. Restricted Yki^S168A^-Mediated Growth Is Associated with Reduced Collagen Expression

**Figure 4:**
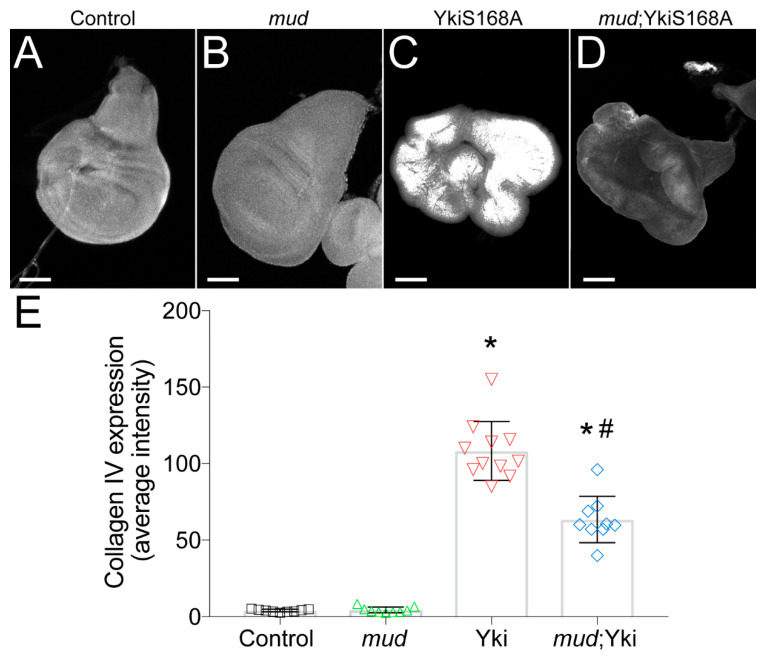
*mud^RNAi^* reduces collagen accumulation stimulated by Yki^S168A^ expression in imaginal wing discs. (**A**–**D**) Type IV collagen staining was used to determine the accumulation of this core BM component in wing discs from each of the indicated genotype. Discs were imaged at identical microscope settings (e.g., gain and laser intensity) for all genotypes. Images shown are of raw images threshold masked according to methods and set to equivalent contrast levels. These images are representative of at least 10 discs. (**E**) Bar graph depicts the average ± standard deviation of collagen measured in average pixel intensity across entire wing disc pouch. *, *p* < 0.05 compared to Control; #, *p* < 0.05 compared to Yki^S168A^ alone, ANOVA with Tukey’s post-hoc test. Note that images are not shown at identical scales. Specifically, scale bars shown represent 50, 50, 250, and 150 microns for Control, *mud*, Yki^S168A^, and *mud*;Yki^S168A^ images, respectively.

To more closely scrutinize the potential role for ECM/BM remodeling in regulating Yki-mediated disc hyperplasia, we immunostained discs with a type IV collagen antibody to determine how Yki^S168A^ and *mud^RNAi^* altered BM protein composition. Expression of *mud^RNAi^* alone did not significantly alter collagen accumulation; however, we found that Yki^S168A^ expression caused dramatically increased levels of this core BM component (Figure 4). Notably, *mud^RNAi^* significantly reduced this elevated collagen level in Yki^S168A^-expressing discs. Thus, the growth dynamics of each genotype correlate with their respective collagen levels, suggesting that ECM/BM remodeling contributes to their size determination.

These results are intriguing considering how the wing disc BM is constructed during larval development. Normally, collagen is synthesized by adipocytes and secreted into the hemolymph by the fat body, as well as from hemocytes, from which it can continuously incorporate into the BM of developing larval tissues distal to this site of production, such as imaging wing discs [63]. In fact, *vkg* knockdown directly in wing disc epithelial cells themselves does not affect the Vkg protein content in the BM. This raises two important and related questions: how does Yki^S168A^ expression in discs lead to elevated type IV collagen accumulation and why does *mud^RNAi^*-induced reduction of ECM-related gene expression in wing disc cells alter this collagen deposition and restrict Yki-dependent tissue growth? As described above, both Yki^S168A^- and Yki^S168A^;*mud*^RNAi^-expressing discs showed differential expression in numerous ECM-related genes (Table 2 and Figure 3). Notably, these changes were unique to each genotype, suggesting that the underlying mechanisms leading to differential collagen accumulation are also distinctive. With respect to Yki^S168A^-expressing discs and the first question posed above, we did not observe changes in collagen genes directly compared to Control. However, among the genes downregulated in these discs was Secreted Protein, Acidic, Rich in Cysteine (SPARC; Table 2). SPARC is a conserved ECM component that acts as a collagen-binding chaperone to control spatial and temporal type IV collagen assembly into the maturing BM [65]. Loss of SPARC in the fat body leads to increased accumulation of an insoluble fibrous collagen network around adipocytes [66], which limits its diffusion to distant assembly sites [63]. More importantly, studies have shown that reduced SPARC expression directly in the follicular epithelium leads to an autonomous increase in Vkg secretion and accumulation necessary for its morphogenic development [67]. Thus, the reduction of SPARC may allow otherwise insufficient or restricted levels of Vkg produced cell autonomously to accumulate in Yki^S168A^-expressing discs. Regardless of the precise mechanism, a link between Yki signaling and increased collagen accumulation has been described in other systems as well. For example, it has been shown in several human epithelial tissues that YAP/TAZ (the orthologous Yki genes in mammals) activation can induce collagen and other ECM component production [68,69,70]. This remodeling of the ECM fortifies focal adhesion and recruits additional collagen-secreting fibroblasts, leading to further mechanotransduction-mediated YAP/TAZ activity in a feedforward loop [71,72,73]. Indeed, several ECM-related genes have been identified as direct targets of YAP/TAZ transcriptional regulation [74,75].

Mud knockdown did not simply reverse the changes seen under Yki^S168A^ expression alone (e.g., an increase in SPARC was not detected), which suggests that a distinctive mechanism is likely at play. That mechanism seems to involve a direct reduction in the *vkg* transcript itself, which would reduce any ability gained by Yki^S168A^ expression in autonomous collagen secretion. Changes in other ECM-related genes may also contribute to this effect (Table 5). How these changes may causally relate to size restriction could involve several mechanisms. For example, recent work in wing discs has shown that an intact BM promotes tissue growth through retention of Decapentaplegic (Dpp; the fly ortholog of BMP/TGF-β), rather than through direct mechanoregulation of the Hippo pathway [64]. Similar links between type IV collagens and BMP signaling have also been shown in other tissues [76,77]. Moreover, several studies have demonstrated cooperative growth promoting effects between BMP and Yki [14,78,79]. Thus, the *mud^RNAi^*-mediated reduction in ECM/BM gene transcript levels and subsequent perturbation of collagen protein accumulation could act through these pathways to restrict Yki-dependent growth and hyperplasia. Another possibility is that Yki^S168A^ expression and Mud knockdown affects hemocyte recruitment or function to indirectly alter collagen deposition and accumulation. Overall, our results suggest that persistent Yki activation may induce direct collagen production, which is impaired following transcriptional alterations induced by Mud knockdown. However, deciphering the exact mechanism for these changes will require future study.

## 4. Conclusions

The elucidation of the genetic mechanisms controlling tissue growth has profound importance in understanding both normal development as well as disease progression. Owing to their ubiquitous expression and disease relevance, epithelial are a particularly important tissue type in this pursuit. Using *Drosophila* wing discs as a model system, we have shown that knockdown of Mud, a core mitotic spindle regulator, limits hyperplastic growth induced by misexpression of constitutively active Yki. Mutations in the Yki pathway led to overgrowth without disruption of tissue architecture by promoting cell proliferation and preventing cell death [80]. Mud loss, in contrast, impeded cell cycle progression and induced elevated apoptosis in Yki-expressing discs to restrict growth. Through transcriptomic analyses, we identified a putative role for ECM/BM modulation in response to both Yki^S168A^ and *mud^RNAi^*, with changes in gene expression potentially leading to altered collagen protein accumulation that ultimately affect wing disc growth [64]. Our work highlights several important future questions to address as well. Are the effects of Mud loss on Yki-mediated growth unique or do other spindle-associated genes (e.g., SAS-4 [36]) have similar interactions? Does Mud loss restrict hyperplastic growth induced by other oncogenes (e.g., EGFR [81]), and how does it impact neoplastic growth typified by others (e.g., *ras^V12^;scrib* [82])? What is the molecular mechanism linking Mud loss to transcriptional changes, and is this effect cell-autonomous? Are these functions conserved in human tissue (e.g., with the NuMA and YAP/TAZ orthologous genes)? These and others questions await further study that should add additional insights into the growth dynamics and tumorigenic potential of epithelial tissue.

The work presented here has broad implications for both normal tissue and organ development as well as tumor suppression. The Hippo/Warts/Yorkie signaling pathway has emerged as a major contributor to tissue development, namely in the control of tissue and organ size determination, and one that is evolutionarily conserved in animals [8]. Deficits in Yki activity lead to retardation in tissue growth and abnormal developmental. In contrast, hyperactivation of this transcriptional regulator can cause hyperplastic overgrowth and has been found in a variety of human tumors [6,28,83]. Yorkie has also been recently shown to cooperate with other tissue growth and morphogenic pathways critical during development, which also influence stem cell maintenance and cell fate decision in diverse tissues [79,84,85]. Recent studies have identified genes that, when disrupted in function, synergistically promote oncogenic transformation during development of tissues with concomitant Yki hyperactivation [11,14,15]. Our findings here showing that disruption of the *mud* function can, in contrast, restrain Yki-mediated hyperplastic overgrowth add important new insights into mechanisms that intersect with this key mediator of tissue development and growth. The requirement for Mud, a microtubule-associated mitotic protein, in Yki-driven growth suggests a critical role for an intact mitotic spindle in maintaining growth. Finally, the unexpected findings suggesting that ECM and BM dynamics may also be important in regulating Yki function add additional insight into the mechanical regulation of tissue development.

## Figures and Tables

**Table 1:** Gene ontology (GO) terms enriched in Yki^S168A^-expressing discs (relative to Control).

**GO Terms Enriched in Yki^S168A^-Expressing Discs (Relative to Control)**	
**Upregulated Genes (Biological Process)**	**−Log_2_*p*-Value**
Cytoplasmic translation	130.2
Cellular process	80.1
Cellular metabolic process	64.9
Single organism cellular process	59.9
Organonitrogen compound metabolic process	54.8
**Upregulated Genes (Molecular Function)**	**−Log_2_*p*-Value**
Structural constituent of ribosome	56.3
Structural molecule activity	25.8
Cofactor binding	23.8
Translation factor activity; RNA binding	15.7
Translation initiation factor activity	13.6
**Downregulated Genes (Biological Process)**	**−Log_2_*p*-Value**
System development	179.7
Regulation of biological process	175.9
Regulation of cellular process	172.9
Biological regulation	172.6
Animal organ development	169.8
**Downregulated Genes (Molecular Function)**	**−Log_2_*p*-Value**
Sequence-specific DNA binding	36.3
Nucleic acid binding transcription factor activity	35.8
Transcription factor activity, sequence-specific DNA binding	35.8
mRNA binding	33.4
RNA polymerase II transcription factor activity, sequence-specific DNA binding	32.6

Gene ontology terms listed, whether biological process or molecular function, represent genes significantly upregulated or downregulated in Yki^S168A^-expressing discs relative to Control discs. −log2 *p* vales are listed.

**Table 2:** Extracellular matrix (ECM)-related genes upregulated or downregulated in Yki^S168A^ discs.

**ECM-Related Genes Altered in Yki^S168A^-Expressing Discs (Relative to Control)**	
**Gene**	**Annotated Function (FlyBase.org)**
***Upregulated***	
*dpy*	ECM protein involved in cuticle attachment
*mmy*	Protein glycosylase involved in ECM and cuticle construction
*wb*	Laminin-α chain ECM protein; BM integrity and integrin signaling
*serp*	Secreted apical ECM protein involved in cuticle construction
*trol*	Perlecan ECM secreted heparan sulfate proteoglycan
*verm*	Chitin deacetylase involved in cuticle development
*Timp*	Inhibitor of matrix metalloproteinases, regulates ECM remodeling
*by*	Tensin, binds integrin/actin to regulate wing surface adhesion
*mgl*	Apical membrane protein, cuticle development
*Ndg*	Structural ECM protein, organizes BM assembly
*Mmp1*	Matrix metalloproteinase, regulates ECM remodeling
*mfas*	Regulator of cell adhesion and hemocyte proliferation
*sona*	ADAM protease in ECM, positive regulator of tissue growth
*mspo*	Spondin family ECM glycoprotein
*Crag*	Rab exchange factor involved in BM protein secretion
*scaf*	Inactive serine protease, regulates Laminin localization in BM
*Gasp*	Regulator of cuticle biosynthesis
*Cpr47Eb*	Structural component of cuticle
*if*	Integrin α-subunit of receptor for ECM
*Itgbn*	Integrin β-subunit of receptor for ECM
*Vinc*	Vinculin; mechanotransducer in cell-matrix interactions
*stck*	Integrin adaptor protein, wing disc apposition
*plx*	RabGAP, regulates integrin-mediated adhesion
*Pax*	Paxillin; integrin adaptor
*Itgbn*	
***Downregulated***	
*Dg*	Non-integrin ECM-actin adapting receptor
*dlp*	Glypican ECM membrane heparan sulfate proteoglycan
*Glt*	Secreted component of BM
*LanB2*	Laminin-B2; BM component interacts with ECM and integrins
*Mmp2*	Metalloproteinase, regulates ECM remodeling
*Mp*	Collagen XV/XVIII member
*Ppn*	ADAM-like protein, regulates ECM construction
*Pxn*	Peroxidase, regulates BM assembly
*scb*	α-PS3 Integrin, ECM ligand function
*SPARC*	Collagen and ECM-binding protein; BM maturation
*Tig*	Integrin ligand component of ECM
*Tsp*	Integrin ligand component of ECM

Notable ECM-related genes identified as statistically upregulated or downregulated in Yki^S168A^-expressing discs compared to Control discs.

**Table 3:** GO terms enriched in *mud^RNAi^*-expressing discs (relative to Control).

**GO Terms Enriched in *mud^RNAi^*-Expressing Discs (Relative to Control)**	
**Upregulated Genes (Biological Process)**	**−Log_2_*p*-Value**
Cytoplasmic translation	69.6
Organic substance biosynthetic process	23.4
Regulation of cellular protein localization	22.5
Biosynthetic process	21.4
Cellular biosynthetic process	21.3
**Upregulated Genes (Molecular Function)**	**−Log_2_*p*-Value**
Structural constituent of ribosome	27.2
Structural molecule activity	21.5
rRNA binding	16.3
Unfolded protein binding	10.1
ATP binding	10.0
**Downregulated Genes (Biological Process)**	**−Log_2_*p*-Value**
Tissue development	26.5
Negative regulation of translation	25.0
Negative regulation of cellular amide metabolic process	23.9
Response to stimulus	23.8
Single-multicellular organism process	21.8
**Downregulated Genes (Molecular Function)**	**−Log_2_*p*-Value**
Nutrient reservoir activity	18.6
mRNA 3′-UTR binding	13.0
Receptor binding	11.4
Translation repressor activity	11.3
Nucleic acid binding transcription factor activity	11.1

Gene ontology terms listed, whether biological process or molecular function, represent genes significantly upregulated or downregulated in *mud^RNAi^*-expressing discs relative to Control discs. −log2 *p* vales are listed.

**Table 4:** GO terms enriched in *mud^RNAi^*;Yki^S168A^-expressing discs (relative to Yki^S168A^ expression alone).

**GO Terms Enriched in *mud^RNAi^*;Yki^S168A^-Expressing Discs (Relative to Yki^S168A^)**	
**Upregulated Genes (Biological Process)**	**−Log_2_*p*-Value**
Apoptotic process	10.5
Programmed cell death	7.9
Regulation of apoptotic process	7.7
Cell death	7.5
Regulation of programmed cell death	7.2
**Upregulated Genes (Molecular Function)**	**−Log_2_*p*-Value**
Double-stranded RNA-specific ribonuclease activity	6.5
Nuclease activity	5.5
Catalytic activity	4.4
**Downregulated Genes (Biological Process)**	**−Log_2_*p*-Value**
Post-embryonic development	12.3
Chitin-based cuticle development	10.4
Striated muscle cell differentiation	9.3
Multicellular organism development	9.2
Anatomical structure development	8.9
**Downregulated Genes (Molecular Function)**	**−Log_2_*p*-Value**
Structural constituent of chitin-based larval cuticle	9.1
Extracellular matrix structural constituent	9.0
Structural molecule activity	8.4
Structural constituent of chitin-based cuticle	8.1
Structural constituent of cuticle	7.7

Gene ontology terms listed, whether biological process or molecular function, represent genes significantly upregulated or downregulated in *mud^RNAi^*; Yki^S168A^-expressing discs relative to Yki^S168A^-expressing discs. −log2 *p* vales are listed.

**Table 5:** ECM-related genes downregulated in *mud^RNAi^*;Yki^S168A^ discs.

**ECM-Related Genes Downregulated in *mud^RNAi^*;Yki^S168A^-Expressing Discs (Relative to Yki^S168A^ Alone)**	
**Gene**	**Annotated Function (FlyBase.org)**
*vkg*	Type IV collagen; core BM component
*Col4a1*	Type IV collagen; core BM component
*tnc*	Structural component of ECM/collagen
*pot*	Transmembrane protein component of apical ECM; links epithelia to cuticle and organizes microtubules
*Glt*	Secreted glycoprotein component of BM
*kirre*	Transmembrane adhesion protein and ECM interactor
*Cpr49Ac*	Cuticle component
*Lcp1*	Structural component of cuticle

Notable ECM-related genes, including collagen, identified as statistically downregulated in *mud^RNAi^*; Yki^S168A^ discs compared to Yki^S168A^ alone discs.

## Supplementary Materials

The following are available online at https://www.mdpi.com/2221-3759/8/4/34/s1. Figure S1: Equally scaled images of third instar imaginal wing discs illustrate effects of Yki^S168A^ and *mud^RNAi^* expression on disc size; Table S1: Raw RNAseq data; Table S2: Lists of all differentially-expressed genes; Table S3: Transcription factor motif analyses.
