# Supplementary material for: Mud Loss Restricts Yki-Dependent Hyperplasia in Drosophila Epithelia
Source: J Dev Biol. 2020 Dec 13;8(4):34. doi: 10.3390/jdb8040034 (PMC7768408; doi:10.3390/jdb8040034)
Supplement: Supplementary file 1 [file jdb-08-00034-s001.zip › jdb-1000457-suppl/Figure S1.pdf]

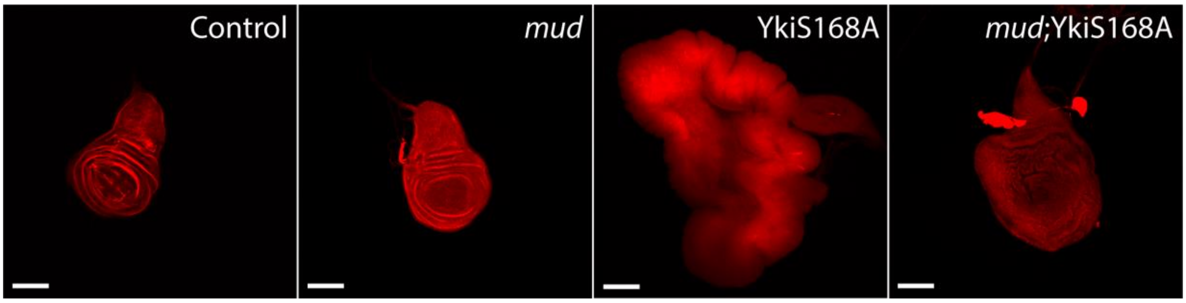

Figure S1. Equally scaled images of third instar imaginal wing discs illustrate effects of Yki^{S168A} and *mud*^{RNAi} expression on disc size
